# Addressing Global Disparities in Lung Cancer Screening: Lessons From Puerto Rico and Beyond

**DOI:** 10.1016/j.jtocrr.2025.100919

**Published:** 2025-10-15

**Authors:** Patrick Goodley, Matthew Evison

**Affiliations:** a Wythenshawe Hospital, Manchester University NHS Foundation Trust, Manchester, United Kingdom; b Faculty of Biology, Medicine, and Health, The University of Manchester, Manchester, United Kingdom

Lung cancer screening (LCS) has been proven to reduce mortality by detecting lung cancer at early stages. It has been available in the United States since its pioneering recommendation by the U.S. Preventive Services Task Force more than a decade ago.^1^ Fundamentally, screening can only benefit people who participate. Despite its early adoption in the United States, LCS uptake rates have lagged behind ambition.

The cross-sectional study by Castañeda-Avila et al.^2^ used data from the Behavioral Risk Factor Surveillance System (BRFSS) survey to explore LCS uptake in the U.S. territory of Puerto Rico.^3^ The BRFSS is the flagship national Centers for Disease Control and Prevention survey relating to health behaviors, which, in 2022, included detailed smoking history questions. This enabled the authors to determine who would be eligible for screening on the basis of the U.S. Preventive Services Task Force 2021 criteria (age 50–80 years, ≥20 pack-year current or recent smoking history). Respondents were grouped into U.S. non-Hispanic, U.S. Hispanic, and Puerto Rico categories. The Puerto Rican cohort was estimated to have lower educational attainment and income, but similar rates of health insurance coverage and recent engagement with health care as the other cohorts. These signals suggest that access to health care exists in general, but screening lags behind other services.

A limitation of this study is its reliance on self-reported screening uptake. The survey design inherently brings a degree of recall error and selection bias, as acknowledged by the authors. Although the BRFSS survey is large, there were only 174 eligible respondents from Puerto Rico from which to extrapolate the study population. This explains the wide confidence intervals (CI) around point estimates, reducing certainty regarding the true extent of differences among these populations of interest. As suggested, future reporting of objective measures of screening provision, such as clinical activity, insurance claims, or registry data, would be of interest to corroborate the survey findings.

The primary comparison was of self-reported LCS uptake among eligible individuals. The authors base their conclusion on the finding that the proportion of Puerto Ricans being up to date with screening (within the past 12 months) was 9.8%, or 54% of the proportion reported among other U.S. residents (adjusted prevalence ratio 0.54, 95% CI 0.29–0.99). This suggests that Puerto Rico has a lower-quality screening provision than the United States, a potential driver of health inequalities. However, the proportion who had undergone screening at any time was similar between cohorts, in the range 28% to 32%, with an adjusted prevalence ratio of 0.89 (95% CI 0.65–1.23). The time since last screening was captured in BRFSS, but was not reported in further detail here nor in other studies on the basis of BRFSS data.^4^^,^^5^

The importance of having annual screening compared with longer screening intervals is debatable. It is arguably more important to have been screened at least once, even if more than 12 months ago, than to be up to date. Screening programs in other settings are offered biennially. Although early detection may be superior at annual intervals, biennial screening is still effective^6^ and is standard practice in other countries such as England and Australia. For the same number of screening computed tomography (CT) scans, the benefit to population health would be greater if more people were screened less often. Therefore, providing screening every 2 years instead of annually is likely to be a reasonable alternative, particularly given health care resource limitations.

In light of this, perhaps the key finding of this study is that screening uptake is low across the United States. Figure 1 starkly illustrates that, across all groups, whether living in Puerto Rico or not, Hispanic or not, approximately 70% of eligible individuals have never been screened. As acknowledged in the discussion, this is likely an underestimate of nonparticipation. Although 100% uptake is neither realistic nor desirable (people are perfectly entitled to opt against screening), 30% uptake surely reflects shortages of awareness and accessibility.

**Figure 1:**
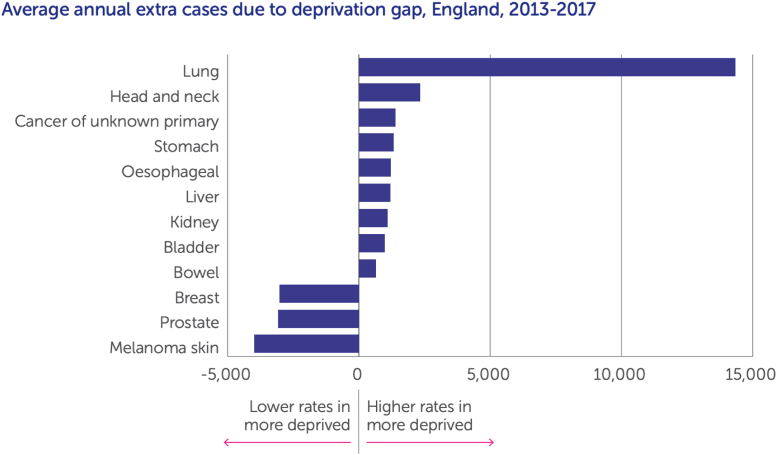
Average annual cancer cases due to deprivation gap in England, 2013 to 2017. Reproduced from Cancer Research UK.^7^

Interpretation of uptake rates must take account of differences in how screening is offered. In the United States, the onus is usually on primary care providers to identify individuals who are at high risk and then offer them screening scans. In the United Kingdom, systematic invitation leads to a risk assessment, and then high-risk individuals are offered screening. Measurement of uptake among the actual eligible population is difficult when the true number of high-risk individuals who do not engage at all is not directly captured and therefore uncertain, so there is a shortage of high-quality international data. Systematic surveys of populations irrespective of attendance at screening, such as in this study, present a valuable way of overcoming this challenge.

More widely, the potential for screening programs to widen or narrow health inequalities must be considered. Disparities are associated with socioeconomic status, health literacy, age, sex, sexual orientation, ethnicity, and disability.^8^ Although there is a shortage of high-quality data from which to disentangle the relative contributions of these often-overlapping factors, some key themes have been identified.

There are numerous potential barriers to uptake, including lack of population invitation, real or perceived financial costs,^9^ convenience of screening sites,^10^ and perceptions of stigmatization and futility.^11^ After screening, there are further opportunities for health inequalities to arise, including availability of and engagement with diagnostics and treatment, and longer-term survivorship care. Such barriers disproportionately disadvantage people at the highest risk of lung cancer who could gain the most from early detection. These risks widen health care inequalities and undermine the population health gains that screening can offer.

However, screening can be a powerful tool to reduce health inequalities. When easily available and followed by effective diagnostic and treatment pathways, screening can bring the benefits of early detection to parts of society that suffer poorer outcomes.

It is worth looking at programs outside the United States to consider potential strategies to improve uptake at each step in the path leading to screening. In England, for instance, systematic invitation to screening is offered in a free universal health care setting, and this has seen uptake rates in the range of 41% to 53%.^12^, ^13^, ^14^, ^15^ Invitation is on the basis of comprehensive primary care registries, and every individual in the population who may potentially be eligible for screening on the basis of age, with or without smoking status, receives an invitation letter. Many of these programs deployed mobile screening units into communities to support accessibility. These participation rates could be further improved on, particularly among people who currently smoke, men, and those with socioeconomic deprivation.

Socioeconomic deprivation is a fundamental driver of health inequality. Mechanisms include higher smoking prevalence and disease incidence, lower health care utilization and screening uptake leading to more late-stage emergency presentations, and ultimately higher mortality. Socioeconomic disparity is particularly stark with lung cancer, as illustrated in Figure 1. Notably, the growing National Health Service (NHS) Lung Cancer Screening Programme was first launched in the most socioeconomically deprived parts of the country, prioritizing these areas, which also suffer the highest lung cancer burden. This has already yielded high levels of early detection in these areas, with 75% of diagnoses at stage I to II,^16^ with national impact already noted^17^ despite only beginning in 2019 and still being only available in less than half the country. Reaching people who currently smoke is a key challenge, particularly as attendance at screening presents an additional opportunity to improve health through smoking cessation support. There are fundamental differences in health care systems among countries. However, some lessons may be learned from such settings, such as the potential advantages of population-wide invitation, centralized programs, and community-based screening locations. After screening, care must be taken to similarly promote equitable access to diagnostics and treatment.

In summary, improving LCS uptake is key to translating the mortality benefit seen in clinical trials into maximal population health benefit. The study by Castañeda-Avila et al.^2^ offers insight into the particular challenges faced in Puerto Rico. Although limitations in self-reported data must be acknowledged, the findings point to a need to refine implementation strategies to improve screening provision in an equitable manner. Innovative approaches to case identification, invitation, and delivery models are key to enhancing uptake and reducing lung cancer mortality at the population level. All the while, it will be important to study health inequalities internationally to identify targets for improvement and evaluate their impact as we seek to provide the highest quality screening to those who could benefit.

## Disclosure

The authors declare no conflict of interest.
